# Evaluation of a content-based image retrieval system for radiologists in high-resolution CT of interstitial lung diseases

**DOI:** 10.1186/s41747-024-00539-w

**Published:** 2025-01-13

**Authors:** Benjamin Böttcher, Marly van Assen, Roberto Fari, Philipp L. von Knebel Doeberitz, Eun Young Kim, Eugene A. Berkowitz, Felix G. Meinel, Carlo N. De Cecco

**Affiliations:** 1 Division of Cardiothoracic Imaging, Department of Radiology and Imaging Sciences, Emory University Hospital, Atlanta, GA USA; 2 Institute of Diagnostic and Interventional Radiology, Pediatric Radiology and Neuroradiology, University Medical Centre Rostock, Rostock, Germany; 3 Clinical and Experimental Medicine PhD Program, University of Modena and Reggio Emilia, Modena, Italy; 4 Institute of Clinical Radiology and Nuclear Medicine, University Medical Center Mannheim, Medical Faculty Mannheim of Heidelberg University, Mannheim, Germany; 5 Department of Radiology, Gil Medical Center, Gachon University College of Medicine, Namdong-Daero 774 Beon-Gil, Namdong-gu, Incheon, South Korea

**Keywords:** Artificial intelligence, Diagnosis (computer-assisted), Lung diseases (interstitial), Tomography (x-ray computed)

## Abstract

**Background:**

This retrospective study aims to evaluate the impact of a content-based image retrieval (CBIR) application on diagnostic accuracy and confidence in interstitial lung disease (ILD) assessment using high-resolution computed tomography CT (HRCT).

**Methods:**

Twenty-eight patients with verified pattern-based ILD diagnoses were split into two equal datasets (1 and 2). The images were assessed by two radiology residents (3rd and 5th year) and one expert radiologist in four sessions. Dataset 1 was used for sessions A and C, assessing diagnostic accuracy and confidence with mandatory and without CBIR software. Dataset 2 was used for sessions B and D with optional CBIR use, assessing time spending and frequency of CBIR usage. Accuracy was assessed on the CT pattern level, comparing readers’ diagnoses with reference diagnoses and CBIR results with region-of-interest (ROI) patterns.

**Results:**

Diagnostic accuracy and confidence of readers showed an increasing trend with CBIR use compared to no CBIR use (53.6% *versus* 35.7% and 50.0% *versus* 32.2%, respectively). Time for reading significantly decreased in both datasets (A *versus* C: 104 s *versus* 54 s, *p* < 0.001; B *versus* D: 88.5 s *versus* 70 s, *p* = 0.009), whereas time for research increased with CBIR software use (A *versus* C: 31 s *versus* 81 s, *p* = 0.040). CBIR results showed a high pattern-based accuracy of overall 73.4%. Comparison between readers indicates a slightly higher accuracy of CBIR results when more than one ROI was used as input (77.7% *versus* 70.1%).

**Conclusion:**

CBIR software improves in-training radiologist diagnostic accuracy and confidence while reducing interpretation time in ILD assessment.

**Relevance statement:**

Content-based image retrieval software improves the assessment of interstitial lung diseases (ILD) in high-resolution CT, especially for radiology residents, by increasing diagnostic accuracy and confidence while reducing interpretation time. This can provide educational benefits and more time-efficient management of complex cases.

**Key Points:**

- A content-based image retrieval (CBIR) software improves diagnostic accuracy and confidence for in-training radiologists for interstitial lung disease (ILD) assessment on computed tomography (CT).
- A CBIR application provides condensed information about similar HRCT cases reducing time for ILD assessment.
- CBIR algorithms benefit from the input of multiple regions of interest per ILD case.

**Graphical Abstract:**

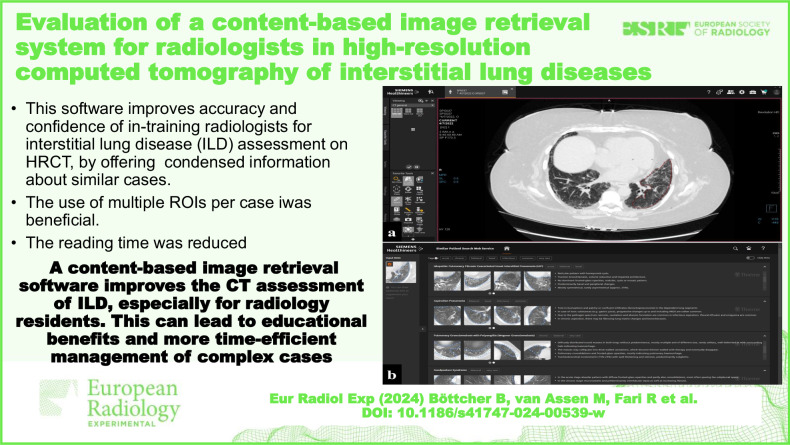

## Background

Interstitial lung diseases (ILD) are very heterogeneous pathologies of the respiratory system in which inflammation and fibrosis cause destruction of lung structure. Classification of these entities is complex and various multidisciplinary guidelines are established to stratify clinical decisions in this field [1–5]. Although improvements were made, defining a precise diagnosis is still challenging, and agreement between multidisciplinary teams is only moderate [6].

High-resolution computed tomography (CT) provides detailed images and is a cornerstone in the diagnostic pathway of ILD [1–5]. Despite new developments in both acquisition technology and post-processing increasing image quality [7–9], discrimination between various ILD patterns is a major challenge with only moderate interobserver agreement, even in experienced radiologists [10, 11].

Content-based image retrieval (CBIR) systems for CT datasets are designed to perform an image-based query to receive similar CT scans from a database. These systems use image information, including but not limited to pattern or attenuation values from a selected input, for example, a region-of-interest (ROI), to identify similar CT images. Additionally, retrieved CT datasets can be supplemented with assigned pathologies and more detailed case-related information to assist radiologists in diagnostic decision-making.

Early investigations showed promising results for CBIR systems used for chest CT scans, however, major limitations, such as low computational power, hampered further development [12]. The rise of artificial intelligence and growing computing resources led to significant progress in CBIR application development focusing on different aspects of chest CT scans such as lung nodule differentiation [13], distinction of lung cancer from atypical tuberculosis [14], or assessment of obstructive lung diseases [15]. In particular, a small number of CBIR systems for ILD assessment on high-resolution CT datasets have been proposed in recent years [16–18]. Validation of such CBIR applications in a clinical setting focuses on the accuracy of retrieved images and on the diagnostic confidence of readers as well as the time needed per case [19–22].

Despite this growing data on the performance of CBIR systems in clinical scenarios, there is no data available on interobserver variability of input ROI placement. This is somewhat remarkable since the ROIs placed by the user are critical for the accuracy of the retrieved images and are not yet standardized. Therefore, this study aims to evaluate an AI-based CBIR software with a special focus on the ROI used as input and on how the variability of ROI placements might impact the accuracy of CBIR software.

## Methods

### Ethical approval and patient selection

This single-center cohort study was approved by the responsible Institutional Review Board (IRB number: STUDY00002503; November 8, 2021), and the need for written informed consent was waived.

High-resolution axial thin slice CT image datasets (slice thickness 0.625–1.5 mm) were selected retrospectively according to the following criteria: (i) patients of ≥ 18 years with (ii) a chest CT scan between 2010 and 2021 with verified pattern-based ILD diagnosis. The following common ILD patterns were included: usual interstitial pneumonia, non-specific interstitial pneumonia, and fibrotic as well as non-fibrotic hypersensitivity pneumonitis. The reference diagnosis was defined and verified patterns based on the radiology report and electronic health records, including multidisciplinary board decisions. Four patients without pathological findings on CT were added. Exams with non-diagnostic image quality were excluded.

### Study design

**Fig. 1:**
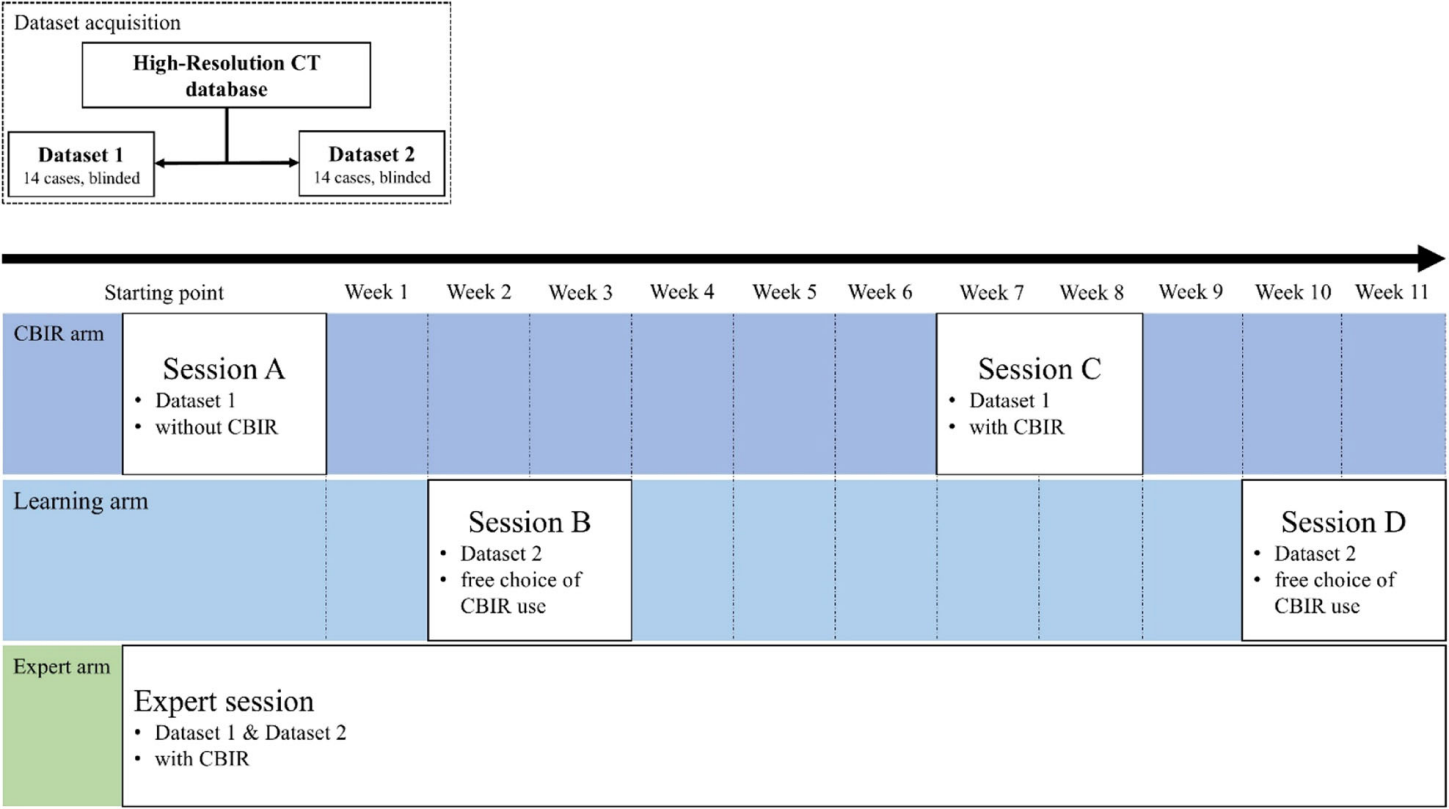
Study design of CBIR software evaluation. First, the retrospectively selected high-resolution computed tomography datasets (*n* = 28) were assigned to one of two datasets (each *n* = 14). Subsequently, sessions from A to D were performed by resident readers at several time points, as visualized above. The expert performed a single session with both datasets. CBIR*,* Content-based image retrieval

The study design is visualized in Fig. 1. Overall, four reading sessions were conducted with a fully blinded case presentation using dataset 1 for sessions A and C and dataset 2 for sessions B and D. In total, 28 CT cases were included and split into two datasets of 14 cases each. Each dataset contained different patients but a similar distribution of cases. Two radiology residents (readers 1 and 2) in their 3rd and 5th year of training (less than one year of chest CT reading experience and two years of experience, respectively) separately performed all sessions and were allowed to change image windowing settings to their preferences and to use digital or non-digital sources of information to assist diagnostic decision-making in all sessions. Since the case presentation was fully blinded, there was no access to medical health records during all sessions. Readers’ diagnostic decisions were given as open answers not limited to the included ILD entities. Prior to the first session, both readers were introduced to the CBIR software package using the user manual and a small number of test cases which were not included in the test datasets.

Sessions A and C aimed to assess diagnostic confidence, diagnostic accuracy, and time needed for image analysis and diagnostic decision-making without (session A, baseline) and with CBIR software use (session C), in the following referred as CBIR arm. Session C was scheduled six weeks after session A to avoid recall bias. Usage of software tools was expected to change with gaining experience and knowledge of software functions and benefits. Therefore, sessions B and D aimed to assess the learning effects of CBIR system use, including time spent on software use and frequency of CBIR software use (learning arm). Each session was scheduled at least one week after sessions A and C. In the learning arm (sessions B and D) readers had free choice to use the CBIR application or not. Additionally, an experienced expert radiologist (+10 years in reading chest CT) performed a separate session (session E) with mandatory CBIR use in each case on both datasets. An independent observer recorded reading times, diagnoses, the confidence of diagnosis, the relevance of software use, results of the CBIR software, and the number of ROIs to ensure a continuous workflow. Feedback on correct or incorrect diagnostic decisions after the sessions was not provided.

### CBIR software

All reading sessions were conducted on a dedicated workstation using commercially available software (syngo.via, version VB50, Siemens Healthineers, Erlangen, Germany) with the fully integrated CBIR application (Similar Patient Search Web Service, version VA41B, Siemens Healthineers, Erlangen, Germany). The CBIR software utilized in this study is founded upon an image embedding function that maps a ROI to a fixed-length feature representation. The embedding function takes the form of a deep residual convolutional neural network, more specifically, a ResNeXt-50 (32 × 4d) [23]. The network backbone was initialized by model weights pretrained on an ImageNet dataset [24]. Input images of the training set were normalized using a mean of 0 and a standard deviation of 1. Model training was performed on a multi-center CT database using metric learning techniques, including triplet loss [25], N-pair loss [26], and an Adam optimizer with a batch size of 256 and 5-fold cross-validation, ensuring that pathological patterns are effectively segregated in the feature space.

**Fig. 2:**
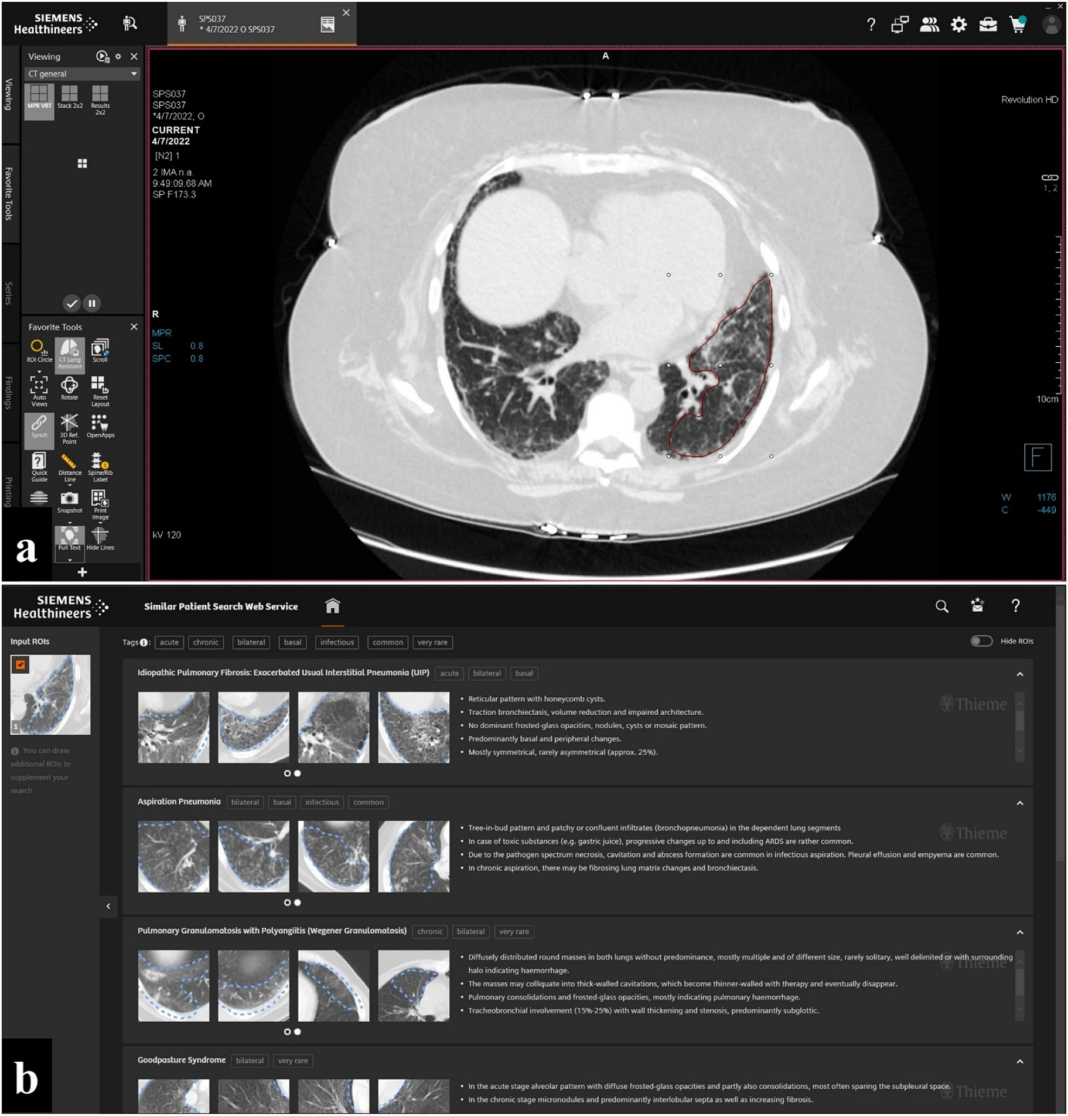
User interface of the content-based image retrieval application (Similar Patient Search Web Service (Siemens Healthineers). **a** Example of an input ROI on an axial high-resolution chest computed tomography in lung window settings. **b** List of retrieved cases with assigned pathologies starting with the most similar at the top. ROI, Region-of-interest

**Fig. 3:**
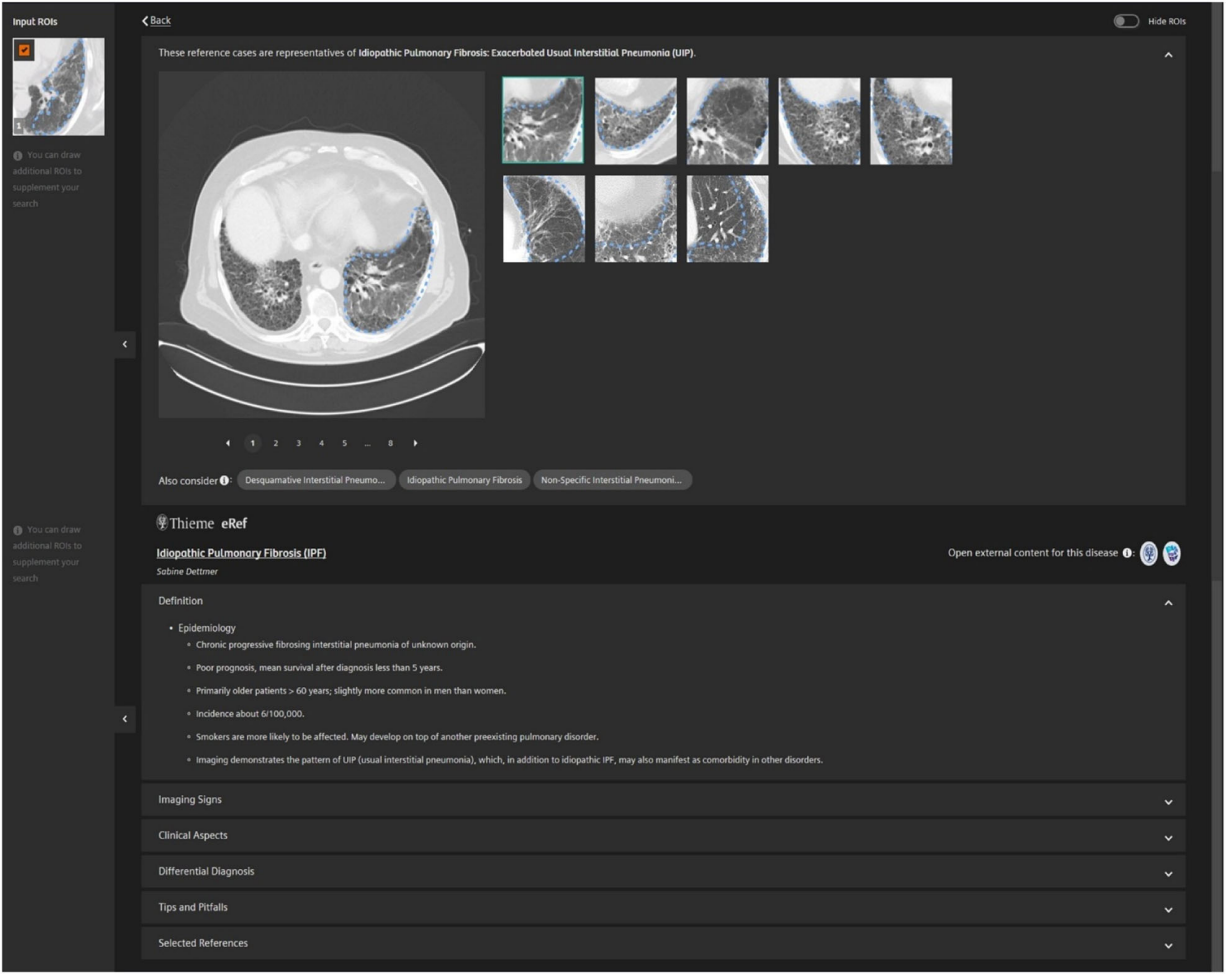
User interface of the content-based image retrieval application after selecting a pathology from the results list. At the top, multiple scans with high similarity to the input ROI can be assessed. Below detailed information of the assigned pathology is provided to assist diagnostic decision-making. ROI, Region-of-interest

A region-of-interest (ROI) based query is used to retrieve cases from a multi-site database of more than 800 CT cases covering 78 ILD diagnoses, see Fig. 2a. The CBIR software allows the use of multiple ROIs simultaneously, and the user can include or exclude individual ROIs from image query manually. Retrieved CT cases are displayed in a list with associated pathologies, starting with the entity with the highest image-based similarity at the top. An example is visualized in Fig. 2b. Each pathology can be selected, and the software provides multiple similar CT scans from its database and additional information, such as definitions, imaging signs, clinical aspects, differential diagnoses, and tips and pitfalls, see Fig. 3.

### Analysis of time, accuracy, and ROIs

Overall time per case was recorded. For detailed analysis, time was measured as time for reading the CT images and research time spent using the CBIR software and/or other sources of information. The accuracy of readers was analyzed by direct comparison between readers’ and verified reference diagnoses (diagnosis-based approach). To assess CBIR software accuracy, verified reference diagnoses and CBIR software results were assigned to characteristic CT imaging patterns (CT-patterns-based approach) by a board-certified radiologist specialized in cardiothoracic imaging (*e.g*., usual interstitial pneumonia was assigned with honeycombing, bronchiectasis, and reticular abnormalities). In addition, each ROI was assigned to an anatomical location and CT pattern. CBIR software accuracy was calculated based on matches between assigned CT patterns of CBIR results and CT patterns within the input ROIs. This pattern-based approach was chosen because the algorithm only had access to image information within ROIs, whereas readers had access to the overall image dataset in each case. The software results were considered accurate if at least one ROI CT pattern was matched.

The objective analysis was performed on overall cases and on per-reader and per-session levels. For per-reader analysis, results from all sessions were evaluated separately for readers 1 and 2 to detect inter-reader variabilities. Per-session analysis was performed to identify changes between sessions A-D by evaluating results from both readers together separated for each session.

### Analysis of diagnostic confidence and CBIR relevance

Readers were asked to scale their confidence in diagnosis on a 5-point Likert scale: 1 = very low confidence in diagnosis; 2 = low confidence in diagnosis; 3 = intermediate confidence in diagnosis; 4 = high confidence in diagnosis; and 5 = very high confidence in diagnosis. Additionally, readers were asked to state if CBIR software was relevant to the diagnostic decision-making process. The software was considered relevant if retrieved images and information helped the reader in the diagnostic decision-making or if confidence in diagnosis was improved.

### Statistical analysis

Wilcoxon signed-rank test was used to evaluate differences in time overall, time used for reading, and time used for research. Differences in the number of ROIs used by each reader were assessed by using the *χ*^2^ test. The McNemar test was employed to investigate discrepancies in diagnostic accuracy and CBIR utilization. Statistical analysis was performed using SPSS statistics, version 28.0.0 (IBM Corporation). Statistical significance was considered for a *p*-value < 0.050.

## Results

### Patient characteristics

The CT database used in this study consisted of 28 high-resolution CT scans from 12 women (42.9%) and 16 men (57.1%) with a mean age of 65 years (range 29–87) and a mean body mass index of 29.9 kg/m^2^. CT imaging was performed at 100 or 120 kVp with automated tube current modulation according to clinical protocols. The slice thickness ranged from 0.625–1.5 mm. CT scans were acquired on 10 different CT scanner models from two different vendors (GE Healthcare and Siemens Healthineers). The database consisted of eight CT scans of each included ILD pattern and four scans without pathological changes.

### Diagnostic accuracy of readers (diagnosis-based)

Over all sessions, readers’ primary diagnosis matched the reference diagnosis in 53/112, 47.3% of cases. In per-session analysis, diagnostic accuracy showed an increasing trend over all sessions from session A (10/28, 35.7%) to session D (15/28, 53.6%, *p* = 0.302) (Table 1).

**Table 1:** Accuracy of primary diagnosis by readers and primary retrieved results by content-based image retrieval software

	Session A *Without CBIR use*	Session B *Optional CBIR use*	Session C *Mandatory CBIR use*	Session D *Optional CBIR use*
Reader				
Overall	53/112, 47.3%			
	10/28, 35.7%	15/28, 53.6%	13/28, 46.4%	15/28, 53.6%
Reader 1	28/56, 50.0%			
	5/14, 35.7%	7/14, 50.0%	8/14, 57.1%	8/14, 57.1%
Reader 2	25/56, 44.6%			
	5/14, 35.7%	8/14, 57.1%	5/14, 35.7%	7/14, 50.0%
CBIR software				
Overall	−	45/61, 73.4%		
		14/18, 77.8%	17/28, 60.7%	14/15, 93.3%
Reader 1		21/27, 77.7%		
Reader 2		24/34, 70.1%		
Expert		16/24, 66.7%		

Reader accuracy was tested against reference diagnosis. CBIR software accuracy was tested against computed tomography patterns within input regions of interest. *CBIR* Content-based image retrieval

On the per-reader level, the accuracy of reader 1 showed an increasing trend with the use of CBIR software from session A (5/14, 35.7%) to session C (8/14, 57.1%, *p* = 0.375). This trend was not observed for reader 2 (5/14, 35.7% to 5/14, 35.7%). The second and third differential diagnoses given by readers showed lower accuracy (second diagnosis 18/112, 16.1%; third diagnosis 24/112, 21.4%) throughout all sessions.

### Diagnostic accuracy of CBIR software (CT-pattern-based)

Accuracy analyses are summarized in Table 1. Primary retrieved cases of the CBIR software matched at least one CT pattern of input ROIs in 45/61 (73.4%) of all cases where CBIR software was used (*n* = 61). The second and third cases retrieved by CBIR showed lower accuracy (second result 21/61, 34.4%; third result 28/61, 46%). Per-session analyses found a significant increase between session B (14/18, 77.8%) to session D (14/15, 93.3%, *p* = 0.031), most likely because of changes in ROI placements. To calculate CBIR accuracy in session C (mandatory CBIR use), normal cases without pathological findings were excluded from analysis because normal cases are not considered by the software.

At per-reader analysis, CBIR software accuracy revealed slight differences between readers 1 and 2 (21/27, 77.7% *versus* 24/34, 70.1%). Primary results of the CBIR software when used by the expert radiologist matched at least one ROI CT pattern in 16/24 (66.7%) of cases.

### Diagnostic confidence

Results of confidence rating on the 5-point Likert scale were grouped into low confidence (score 1 or 2), moderate confidence (score 3), and high confidence (score 4 or 5). A comparison of sessions A and C showed an increasing trend of confidence levels from low to moderate. Confidence in sessions B and D was similar but higher compared to sessions A and C. This might be caused by the fact that CBIR results and reader´s diagnosis mismatch and, therefore, decrease confidence if CBIR use is mandatory. The expert radiologist had a higher confidence consistently throughout all sessions. Confidence ratings are displayed in Table 2.

**Table 2:** Subjective analysis of confidence in diagnosis and relevance of content-based image retrieval software results

	Session A *Without CBIR use*	Session B *Optional CBIR use*	Session C *Mandatory CBIR use*	Session D *Optional CBIR use*
Confidence in diagnosis				
Low (score 1 or 2)	13/28, 46.4%	3/28, 10.7%	8/28, 28.6%	4/28, 14.3%
Moderate (score 3)	6/28, 21.4%	11/28, 39.3%	11/28, 39.3%	10/28, 35.7%
High (score 4 or 5)	9/28, 32.2%	14/28, 50.0%	9/28, 32.1%	14/28, 50.0%
Relevance of CBIR result for diagnostic decision				
Overall	−	32/61, 52.5%		
		11/18, 61.1%	11/28, 39.3%	10/15, 66.7%
Reader 1		15/27, 55.6%		
		3/6, 50.0%	6/14, 42.9%	6/7, 85.7%
Reader 2		17/34, 50.0%		
		8/12, 66.7%	5/14, 35.7%	4/8, 50.0%
Expert		27/28, 96.4%		

CBIR software was considered relevant if retrieved images and information helped the reader in the diagnostic decision-making or if confidence in diagnosis was improved. *CBIR* Content-based image retrieval

### Relevance of CBIR software

Table 2 shows the results of CBIR relevance analysis. CBIR software was rated relevant for diagnostic decision-making in 32/61, 52.5% of all cases with CBIR use (sessions B, C, and D). Per-session analysis showed that CBIR software was considered relevant for the diagnostic decision-making in more cases in sessions with optional CBIR use (session B 11/18, 61.1%; session D 10/15, 66.7%) compared to session C with mandatory CBIR use (11/28, 39.3%).

In per-reader analysis, reader 1 rated CBIR software relevant for diagnostic decisions in 15/27 (55.6%) of cases over all sessions. Reader 2 rated CBIR system relevance slightly lower with 50% of all cases. In contrast, the expert radiologist rated CBIR software results relevant for diagnostic decision-making in 27/28 (96.4%) of all cases, which was unexpected given that the greatest benefit of CBIR use was anticipated for resident readers. This discrepancy may be attributed to disparate interpretations of the retrieved CT cases and associated pathologies, as it can be assumed that in-training radiologists with less experience may encounter greater challenges in interpreting CBIR results compared to expert readers.

### CBIR software use

The use of CBIR software was not allowed in session A and was mandatory in session C. In session B and D readers had free choice to use CBIR software during assessment. The CBIR application was used in 18/28 (64.3%) of cases in session B and in 15/28 (53.6%) of cases in session D. Readers used the CBIR software once per case, multiple uses in the same CT dataset were technically allowed but was not observed during all sessions.

Per-reader analysis showed that CBIR software use of reader 1 slightly increased from session B (6/14, 42.9%) to D (7/14, 50%), whereas software use of reader 2 showed a decreasing trend (12/14, 85.7% *versus* 8/14, 57.1%, *p* = 0.125). This deviation in CBIR use may be caused by different ratings of the relevance of CBIR results for diagnostic decision-making, as reader 2 perceived CBIR relevance slightly lower compared to reader 1. It can be assumed that a decreased relevance of CBIR application results for diagnostic decision-making results in a reduction of CBIR software use.

### Time analysis

Table 3 presents time analysis on a per-session level. Time for reading decreased between subsequent sessions in both study arms (session A, 104 s *versus* session C, 54 s, *p* < 0.001; session B, 88.5 s *versus* session D, 70 s, *p* = 0.009). In the CBIR arm, time for research increased with CBIR software use (session A, 31 s *versus* session C, 81 s, *p* = 0.040). The overall time needed per case showed no significant difference between sessions A (166 s) *versus* session C (145 s, *p* = 0.356), whereas it decreased between session B (159 s) *versus* session D (102.5 s, *p* = 0.006) when CBIR use was optional.

**Table 3:** Interpretation time analysis on the per-session level

	Time overall	Time for reading	Time for research
Session A Without CBIR use	166 s (112–231.5 s)	104 s (81.5–148.5 s)	31 s (0.0–92.3 s)
Session B Optional CBIR use	159 s (74.3–257.0 s)	88.5 s (64.3–154.3 s)	55.5 s (0.0–114.0 s)
Session C Mandatory CBIR use	145 s (89.3–201.0 s)	54 s (37.5–91.0 s)	81 s (41.5–121.8 s)
Session D Optional CBIR use	102.5 s (44.0–190.0 s)	70 s (33.5–111.3 s)	31.5 s (0.0–81.3 s)

Times are displayed in seconds (s) as median (interquartile interval). Time overall = time used from opening the case to set final differential diagnoses, Time for reading = time used for image assessment. Time for research = time used for searching in digital or non-digital sources of information and CBIR software use. *CBIR* Content-based image retrieval

### ROI subanalysis

**Fig. 4:**
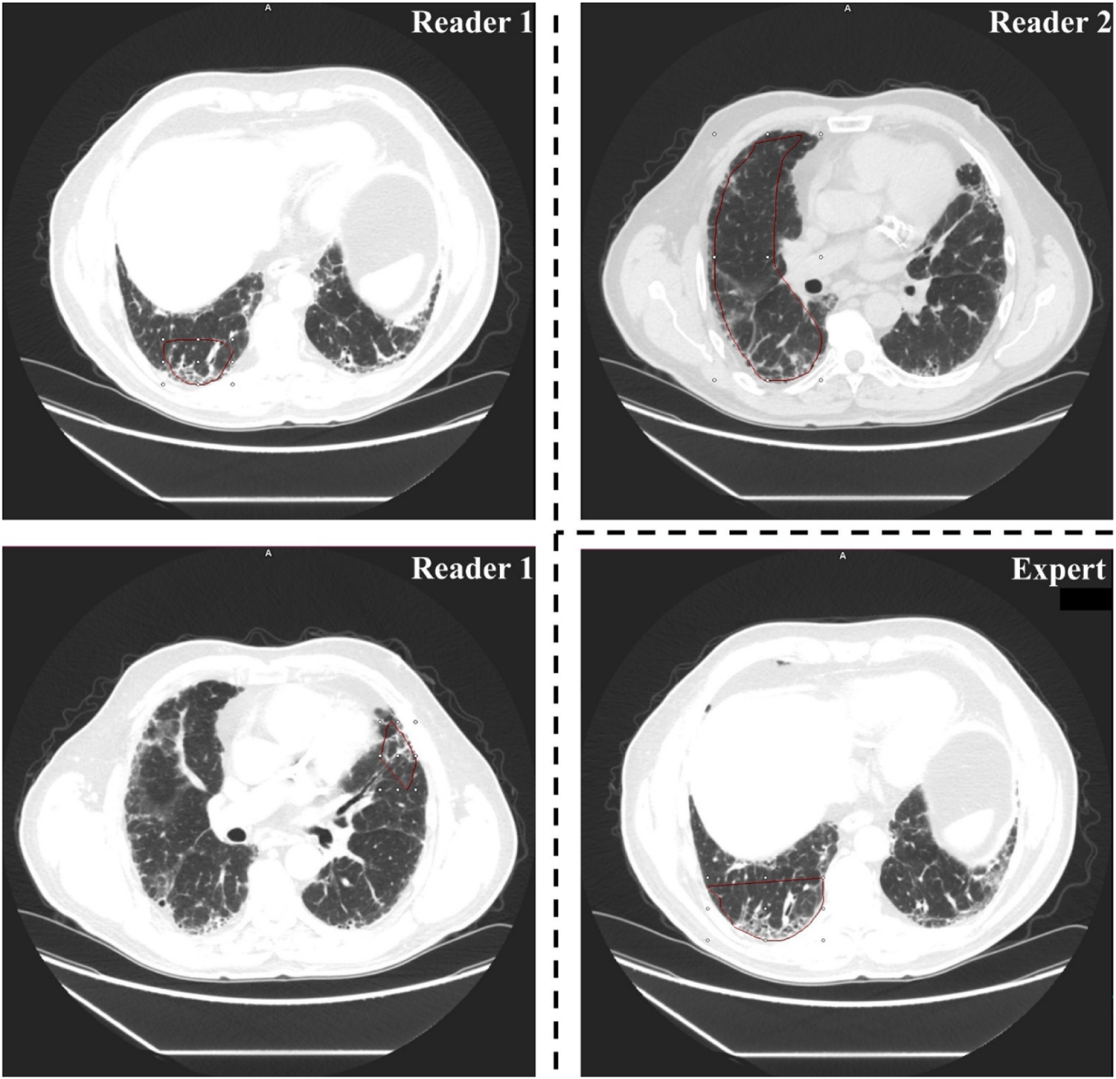
Examples of ROIs used by different users in an identical scan. These images show axial high-resolution computed tomography slices of a patient with an usual interstitial pneumonia pattern. Left: ROIs placed by reader 1. Upper right: ROI placed by reader 2. Bottom right: ROI placed by expert radiologist. ROI, Region-of-interest

Overall, 124 ROIs were placed during all sessions, a mean of 1.4 ROIs per patient. Figure 4 visualizes the ROIs used by readers and the expert in an identical case. Per-reader analysis showed different ROI approaches between readers 1 and 2. Reader 1 used a multiple-ROI approach with a mean of 2.26 ROIs per patient, whereas reader 2 used a single-ROI approach with a mean of 1.06 ROIs per patient (*p* < 0.001). The expert used a single-ROI approach with 1 ROI per case throughout all cases. Reader 2 placed a majority of ROIs in central and peripheral regions (32/35, 91.4%). Reader 1 and the expert used ROIs with peripheral distribution in 35/61, (57.4%) and 13/28 (46.4%) of cases, respectively. They used ROIs with central and peripheral distribution in only 26/61 (42.6%) of cases for reader 1 and 13/28 (46.4%) of cases for the expert reader.

## Discussion

This study evaluated the impact of CBIR software on diagnostic accuracy, confidence, and interpretation time for in-training radiologists with a special focus on interobserver input variability. Results demonstrated an increasing trend of diagnostic accuracy and confidence with the use of the CBIR software. Time spent on CBIR application use decreased with a growing number of use cases. Finally, analysis of input ROIs indicated the beneficial impact of multiple ROIs on the accuracy of the CBIR system.

The accuracy of readers showed an increasing trend with CBIR software use (35.7% *versus* 53.6%, *p* = 0.302), indicating its utility, particularly in low-experienced users. Pogarell et al [20] investigated a similar CBIR software showing improved diagnostic accuracy with CBIR use for students (30% *versus* 85.3%) and residents (60.7% *versus* 93.3%). A recent study by Haubold et al [22] reported for the same CBIR application a significant increase in diagnostic accuracy of residents with CBIR use (18.4% *versus* 33.6%). In contrast to our study design, readers in their study were not allowed to use any additional source of information for ILD assessment during the first session without CBIR application use. This may explain the relatively low increase of accuracy in our study compared to Haubold et al [22]. Additionally, Pogarell et al [20] and Haubold et al [22] included a great variety of ILD entities which can be expected to lead to a greater impact of CBIR use. Röhrich et al [21] evaluated a CBIR application including residents and attending radiologists. Their study also showed an increasing diagnostic accuracy with CBIR software use (34.7% *versus* 42.2%). The CBIR software retrieval output evaluated by Röhrich et al [21] was CT pattern-based, whereas the application investigated in our study provided a disease-based retrieval output. These discrepancies in CBIR results output format may cause differing interpretations by radiologists impacting diagnostic accuracy. Choe et al [19] reported a positive trend of diagnostic accuracy using an in-house developed CBIR (46.1% *versus* 60.9%), including radiology and non-radiology residents, whereas our study focused on radiology residents. This and the lower number of ILD entities in our study might explain differing absolute accuracy values. Overall, the findings of our study and of previous published work suggest that CBIR applications lead to improved diagnostic accuracy of radiologists assessing ILD on chest CT. The fact that diagnostic accuracy was improved even when attending radiologists where included as in the study of Röhrich et al [21] indicates that CBIR applications are also valuable tools for more experienced users. Interestingly, the expert reader in our study perceived a high relevance of CBIR results in 96.4% of all cases, which supports this hypothesis.

In our study, CBIR accuracy showed that in 73.4% of cases, software results matched CT patterns of input ROIs. The database of the CBIR application evaluated in our study does not include normal cases and, therefore, requires sufficient discrimination between normal and pathologic CT cases by users. Radiology residents in our study showed 100% accuracy in detecting normal cases. This indicates that false positive results of CBIR software, when used in non-pathological CT datasets, are a minor risk since these cases can be expected to be detected by the user correctly. Choe et al [19] reported a software accuracy of 80% for matching the same disease class as the query CT, while Hwang et al [16] investigating the same algorithm showed an accuracy of 93.3% for the first retrieved case. Both Choe et al [19] and Hwang et al [16] used CT cases from the CBIR database for image query, whereas our study uses a fully independent dataset. This may cause discrepancies in CBIR accuracy values compared to our study since machine learning algorithms perform better in already-seen cases. Further, the CBIR algorithm used by Choe et al [19] and Hwang et al [16] assessed the whole lung volume. It remains unclear if whole lung assessment leads to improved accuracy because external testing of the algorithm from Choe et al [19] and Hwang et al [16] is missing. The software used in our study uses ROI-based input volumes potentially increasing user variability and error. Further research is needed to explore whether there are relevant differences in retrieval accuracy of CBIR applications using the whole lung volume compared to the ROI-based approach.

ROI analysis in our study showed that readers 1, 2, and the expert used 2.3, 1.1, and 1.0 ROI per patient, respectively. CBIR pattern accuracy was 77.7% (reader 1), 70.1% (reader 2), and 66.7% (expert), indicating an increasing trend of accurate pattern-based retrievals with an increasing number of ROIs per case. ROI use variability in terms of size and location could explain the differences between the CBIR accuracy of reader 2 and the expert. In most cases, reader 2 included peripheral and central lung volume together in relatively large ROIs, whereas the expert tends to do the same in only about half of the cases (91.4% *versus* 46.4%). These results indicate that CBIR software accuracy tends to increase with a growing number and increasing size of ROIs, maximizing the input information.

Time analysis in our study showed an increase in time for research per case when the CBIR software was introduced. However, in subsequent sessions, this time tended to decrease. Time overall per case decreased significantly in subsequent sessions with optional CBIR use. Haubold et al [22] reported an initial increase in time per case of +34% observed during the first half of CBIR use cases which significantly decreased to +7% in the second half. These findings are in line with the decreasing time trends observed in our study. Röhrich et al [21] reported similar results, showing a decrease in overall time used per case of 31% in readings with CBIR. These results support our hypothesis of a significant learning process for users with growing experience in CBIR software use. Like ROI placements, it can be assumed that users are improving the handling of CBIR applications in a reasonable number of use cases, leading to an overall decrease in ILD case interpretation times.

Considering the beneficial impact of CBIR applications on diagnostic accuracy and confidence of radiologists in ILD assessment on chest CT, coupled with an expected favorable learning curve in software use, clinical implementation of CBIR systems for daily use seems feasible. In addition, CBIR systems may be suitable for radiology training programs targeting attending radiologists in private practice or medical students to facilitate the acquisition of knowledge and skills in this field.

This study has several limitations that need to be mentioned. First, our case collection included only a low number of ILD entities with a potential impact on accuracy analysis and power for getting statistical significance. Further, we defined an independent reference standard for the pattern-based accuracy analysis. It is undisputable that ILDs include numerous pathologies, however, a not negligible number of ILD entities are very rare. This is why we focused on the most common pathologies, representing most differential diagnoses in clinical routine. Second, our study provides only a very limited number of in-training readers and a single expert reader who performed the reading sessions in rather small CT datasets. This might have led to a limitation of CT patterns included in the evaluation in terms of severity and variability of occurrence. Further studies with a larger number of readers may be necessary to validate our findings and to explore the full extent of ROI variability’s impact on CBIR accuracy.

In conclusion, the use of CBIR software has been demonstrated to improve diagnostic accuracy and confidence in in-training radiologists while reducing interpretation time in ILD assessment.

## Data Availability

The datasets used and/or analyzed during the current study are available from the corresponding author upon reasonable request. The source code of the evaluated content-based image retrieval system is not accessible to the public.

## Abbreviations

CBIR: Content-based image retrieval
CT: Computed tomography
ILD: Interstitial lung disease
ROI: Region-of-interest
